# Generating Protein Folding Trajectories Using Contact-Map-Driven Directed Walks

**DOI:** 10.1021/acs.jcim.3c00023

**Published:** 2023-03-30

**Authors:** Ziad Fakhoury, Gabriele C. Sosso, Scott Habershon

**Affiliations:** Department of Chemistry, University of Warwick, Coventry CV4 7AL, United Kingdom

## Abstract

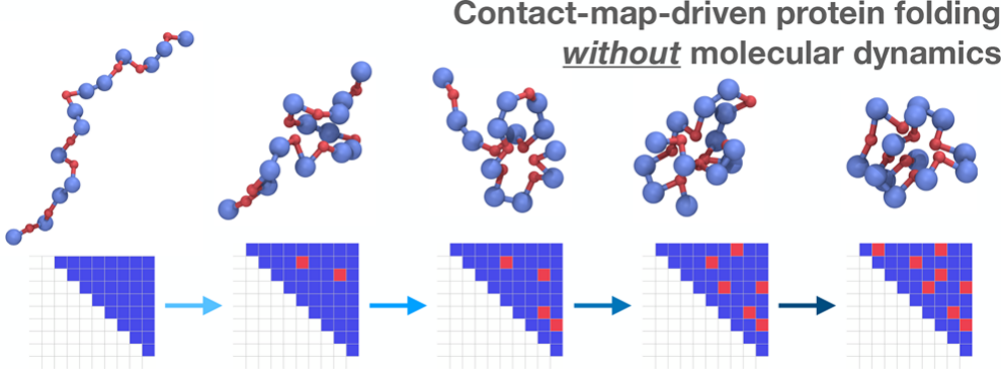

Recent advances in machine learning methods have had a significant impact on protein structure prediction, but accurate generation and characterization of protein-folding pathways remains intractable. Here, we demonstrate how protein folding trajectories can be generated using a directed walk strategy operating in the space defined by the residue-level contact-map. This double-ended strategy views protein folding as a series of discrete transitions between connected minima on the potential energy surface. Subsequent reaction-path analysis for each transition enables thermodynamic and kinetic characterization of each protein-folding path. We validate the protein-folding paths generated by our discretized-walk strategy against direct molecular dynamics simulations for a series of model coarse-grained proteins constructed from hydrophobic and polar residues. This comparison demonstrates that ranking discretized paths based on the intermediate energy barriers provides a convenient route to identifying physically sensible folding ensembles. Importantly, by using directed walks in the protein contact-map space, we circumvent several of the traditional challenges associated with protein-folding studies, namely, long time scales required and the choice of a specific order parameter to drive the folding process. As such, our approach offers a useful new route for studying the protein-folding problem.

## Introduction

Proteins are polymeric biomolecules that underpin the numerous physicochemical operations of biological cells. Naturally occurring proteins fold robustly and reproducibly to their “native state” (i.e., minimum Gibbs free energy conformation), typically over millisecond-to-second time scales.^1^ However, due to the extremely large number of degrees-of-freedom, even for relatively small proteins with a few tens of amino acid residues, the speed of protein folding is considered to be paradoxical.^2^ As such, understanding (and, ultimately, predicting) the folding mechanism for a given protein stands as one of the most challenging problems in biology.

Recently, a major breakthrough was made in protein structure prediction by Alphafold2,^3^ which employed deep learning strategies to predict native protein structures, as demonstrated in the recent Critical Assessment for Structure Prediction (CASP14) competition.^4^ However, while accurate prediction of the final folded protein structure is an invaluable tool in further understanding protein functionality in biological systems, solely predicting the folded state does not offer insights into the folding process itself. Importantly, the thermodynamic and kinetic characteristics of protein folding pathways, as well as the intermediate structures formed, can offer key insights to understand protein folding dynamics, and why folding might fail - as has been implicated in neurodegenerative diseases.^5^

Experimentally, it is extremely challenging to perform a comprehensive study of full protein-folding pathways and their intermediate structures, predominantly because typical folding intermediates are short-lived relative to experimentally accessible time scales for large biological systems. Therefore, it is natural to consider computer simulations as an alternative route to studying protein folding, offering a route to accessing detailed, molecular-level insights into intermediate structures, thermodynamics, and kinetic characteristics of the folding ensemble for a given protein.

The most direct approach to modeling protein folding, namely, molecular dynamics (MD) simulations, has been previously used to study some small, fast-folding (e.g., millisecond time scale) proteins.^6^ Unfortunately, these direct MD simulations often require specially designed high-performance computers that are typically not accessible for most research groups. Even with abundant resources and computational time, many biologically important proteins are much larger than those which have been directly modeled by MD to date and fold over longer time scales than currently accessible on standard hardware. For example, the specially designed supercomputer ANTON2 can perform MD simulations of the ApoA1 protein for periods of 59.4 μs each day;^7^ this is an impressive achievement, but the requisite specialized hardware and software does not offer a generally accessible computational solution for MD simulations of protein folding.

As a result of the “rare event” challenges associated with direct MD simulations of protein folding, many different enhanced sampling computational methods have been developed and employed. For example, successful attempts have been made in accelerating MD simulations by modifying the dynamics of the system to speed up jumps over large activation-energy barriers between different states while preserving their relative rate constants. Representative methods include parallel-replica MD,^8^ hyperdynamics,^9^ and temperature-accelerated MD.^10^ However, an inherent problem in these methods is the “small barrier problem”,^11^ where the accessible speed-up is limited by the fastest kinetic process, such that these simulations can spend too long jumping between relatively shallow minima on the PES.^12^ In the context of protein folding, considering the rugged nature of the Potential Energy Surface (PES),^13^ the assumption that the time scales for jumping between different relevant states are homogeneous is one that is not easy to justify.

Alternatively, one can instead tackle the protein-folding time scale problem by sampling the transition path ensemble. Many popular methods use this strategy and are not limited to Transition Path Sampling (TPS),^14^ Forward Flux Sampling,^15^ and Transition Interface Sampling.^16^ The underlying idea of these methods is to sample several paths between folded and unfolded protein states using a Monte Carlo sampling procedure to generate an ensemble of paths that can be further analyzed for mechanistic and kinetic information. In the context of protein folding, there are significant challenges in applying TPS and its variants. For example, an initial path needs to be provided as a starting point for further sampling, which may be difficult to identify given the complexity of protein folding. Second, and more importantly, TPS will still suffer from rare event sampling problems in “path space”, where a hop from one basin of folding paths to another is computationally inaccessible due to the high degree of correlation in the MC sampling of paths.^17^ In the context of protein folding, where proteins can exhibit multiple folding mechanics and pathways,^18^ this is an important drawback.

A further promising technique that does not suffer from the disadvantages of either accelerated MD or TPS is discrete path sampling (DPS).^19^ This utilizes an “on-the-fly” generated database of local minima and transition states to generate candidate pathways between two configurations of interest, such as folded and unfolded protein structures. Rates of transition between different minima can be evaluated using standard transition-state theory (TST),^20^ and paths with the lowest overall rates can be identified as being significant. The major drawback of this method is that the space of possible local minima, and consequently different paths, is huge, especially when considered in the context of protein folding.

In this article, we introduce and validate a new approach to generate ensembles of protein-folding paths and identify the most physically relevant ones. The key idea underlining this approach is to represent protein-folding trajectories in a discretized representation in which folded and unfolded protein configurations appear as clearly defined states. The generation of protein-folding paths can then be viewed as a problem in discrete optimization, namely identifying directed walks and discrete sequences of transitions that lead from unfolded to folded protein states (which can now be determined with protein structure prediction methods such as Alphafold2^3^). Fortunately, in the case of proteins, it is straightforward to identify a useful discretized space that captures the folding trajectory characteristics. In particular, a residue contact map (i.e., the 2D matrix summarizing whether pairs of residues are in contact) is such a representation, since it encodes the secondary and tertiary structure information on a given protein configuration. Furthermore, the utility of a contact-map as a discrete space of protein trajectories has been successfully demonstrated in recent postanalysis of dynamical behavior during protein folding,^21^ as well as a Markov State Modeling Study where it was shown that structural grouping based on contact maps corresponded well with kinetic accessibility.^22^ This indicates that the contact map is a suitable level of coarse-graining for protein folding and is thus a promising representation to utilize in our approach. We also note that if the study of a particular protein folding pathway required a finer discrete representation, our framework can be extended to account for this, provided there is a set of elementary moves that can be associated with that representation.

With a discrete representation of protein-folding paths available in the form of residue-level contact maps, we are left with two challenges: (i) generation of protein-folding trajectories within the discrete contact-map space and (ii) assessment and ranking of different discrete folding trajectories with regard to their thermodynamic and kinetic characteristics. Recently, in the context of chemical reaction network exploration and catalysis, we have shown how the first of these challenges can be addressed within a discrete optimization task.^23−25^ In particular, our group’s double-ended graph-driven sampling (GDS) approach^25^ employs a discrete bonding-graph representation of a chemical reaction system in order to drive exploration of reaction mechanisms connecting predefined reactant and product configurations. In this article, we build on the GDS approach to study protein folding, by replacing the chemical-bond-based graph used in GDS with a residue-level contact-map description of protein structure. Specifically, we have expanded the scope of GDS to generate sequences of contact-map transformations that construct a direct-walk from a given initial protein structure (i.e., an unfolded protein configuration) to a final target structure (i.e., a folded configuration). As we show below, trajectories generated in contact-map space can be subsequently “back-transformed” into Cartesian protein configurations,^25−27^ enabling direct evaluation of the thermodynamic and kinetic characteristics of each folding-step using standard approaches in reaction-path analysis, such as the nudged elastic band (NEB) method.^28−30^ Importantly, we note that the GDS approach completely circumvents the well-known time scale problems associated with direct MD simulations, and does not require definition of collective variables to “drive” the folding process. In fact, the only prerequisite is the ability to identify the target folded structure by some suitable metric such as radius-of-gyration, contact-map, or coordination number; the flexibility in target definition is a strength of our approach that is discussed later.

In this article, we validated the use of GDS for generating reasonable pathways by comparing the proposed folding paths to those generated by direct MD simulations. Given the time scales associated with protein folding, and the challenges with direct MD simulations of protein-folding noted above, we focus on simulations of so-called off-lattice “HP” protein models comprising hydrophobic (H) and polar (P) residues. For three model proteins of increasing size, we performed detailed comparison of GDS-generated folding trajectories to those available from MD simulations. Analysis using Frechet distance metrics,^31^ trajectory alignment, and multidimensional scaling^32,33^ clearly demonstrates that the set of GDS folding trajectories is representative of the folding trajectories generated by brute-force MD. Furthermore, we also show that the physical feasibility of GDS-generated folding trajectories can be directly assessed and ranked without the need for comparison to a corresponding MD trajectory. This opens the door to wider independent application of GDS to study long time scale protein folding processes. Of course, the GDS method proposed here is not without its own challenges, notably computational expense due to NEB simulations and “back transformation” from contact-maps to residue coordinates, but we also suggest later how these might be addressed.

Finally, we emphasize that the focus of this article is the initial demonstration and validation of GDS as a new scheme for generating protein-folding pathway ensembles; as such, we do not explicitly seek to optimize our simulation approach or related parameters, instead noting avenues for further work as appropriate below. As described below, this validation hinges on comparison to a MD-generated ensemble of folding paths. As a result, we are implicitly limited to studying systems for which we can reliably use MD to generate a large collection of representative folding paths. This means that our validation study here focuses on systems with up to *N* = 34 residues (or beads); we have found that, for the protein model studied here, reliable MD folding with larger proteins (e.g., *N* = 55) becomes much more challenging. However, as shown below, we find that GDS can easily generate and rank folding ensembles for the proteins considered here, opening the door to further applications to larger proteins and more accurate force fields in future work.

## Theory

In this section, we describe our approach to contact-map-driven generation of folding trajectories. First, we outline the simple off-lattice HP model used to describe interactions between residues in the proteins studied here. Next, we show how our GDS method, previously used for chemical reaction mechanism generation,^25−27^ can be adapted to generate plausible pathways leading to a given folded structure. Finally, we describe our approach to validating GDS-generated folding paths. In particular, we show how to compare discrete GDS-generated folding paths to folding trajectories obtained directly by constant-temperature MD simulations. We also show how we can rank the physical feasibility of GDS folding paths without requiring reference to MD trajectories.

**Figure 1:**
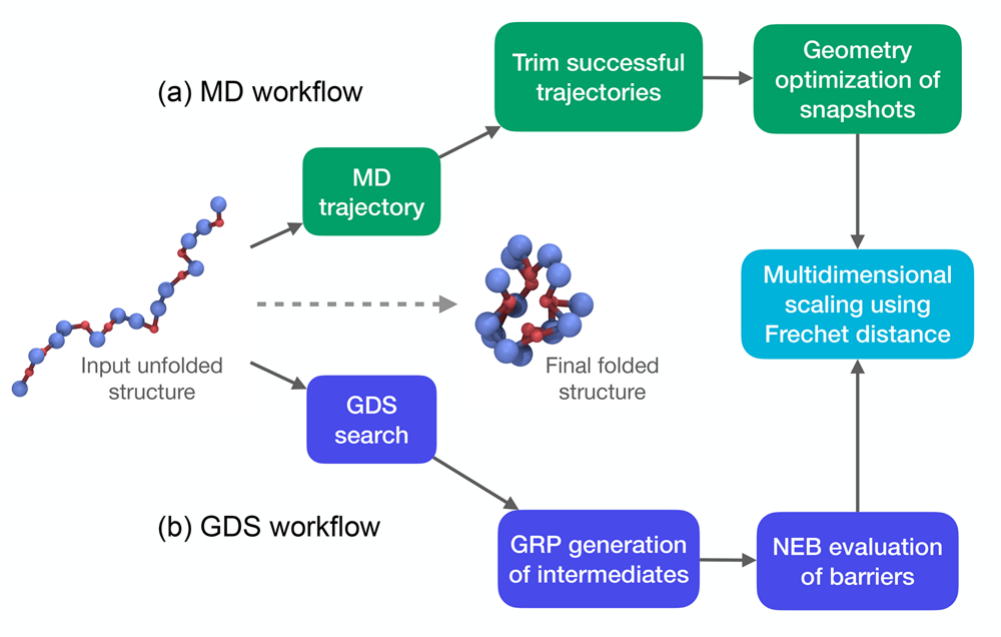
Schematic representation of two different workflows employed in this article. (a) MD workflow, employing MD trajectory generation, trajectory trimming, and geometry optimization of periodic snapshots. (b) GDS workflow, combining GDS path optimization, intermediate structure generation, and NEB MEP calculations. As described below, the folding ensemble generated by GDS can be compared and validated against MD based on Frechet distance.^31^ The representation of the protein here is a hydrophobic–polar representation, with red being hydrophobic and blue polar.

The two different methodological workflows compared here, namely, MD and GDS, are illustrated schematically in Figure 1. As discussed in the following sections, both methods begin with an initially unfolded protein structure. Our key aim is to generate protein-folding trajectories using both MD and GDS, then compare the trajectories to validate the physical accuracy of the folding ensemble obtained by GDS. In our MD workflow (Figure 1a), we first generate a folding trajectory in the canonical (*NVT*) ensemble, after which we trim the trajectory once it reaches the target folded state (removing the long-time part of the MD trajectory which does not contain further information about the folding dynamics). As described below, the MD trajectories generated in this way can subsequently be compared to the GDS-generated trajectories. However, we find that this comparison is typically “contaminated” by the thermal fluctuations of all residues (beads) in the protein. To remove these artifacts, and to enable more straightforward comparison to GDS, we generate MD folding-intermediate structures by periodic geometry optimization of MD snapshots. This transforms the MD trajectory (containing structural changes due to both protein folding *and* thermal fluctuations) into a set of configurations representing conformational changes along a folding path connecting discrete local PES minima. This is much more aligned to the viewpoint of GDS, enabling more straightforward comparison.

Our GDS workflow follows a different path from the MD simulation process (Figure 1b). Here, as described below, we use GDS to generate a folding trajectory, represented as a series of “hops” between discrete folding intermediates. NEB calculations are subsequently performed for each transformation connecting each pair of intermediates along a GDS-generated folding pathway, providing thermodynamic information about each candidate folding trajectory that can be subsequently used to assess and rank different GDS folding paths for physical validity (as described below).

Finally, the two different workflows to generate protein-folding trajectories (MD and GDS) will ultimately be cross-compared as a route to validating GDS. In particular, one would expect that the “best” GDS folding paths are those that most represent the ensemble of MD folding paths. Later, we show how Frechet distance can be used to compared MD and GDS trajectories (Figure 1), but we also demonstrate how GDS can be used to generate and rank folding trajectories using NEB-generated kinetic information alone, without reference to MD trajectory data.

All MD and GDS simulations in this article, as well as their associated analysis, employed custom software written in Fortran and Python with use of the Scipy^34^ library.

### Protein Model Description

Throughout this article, we employ the off-lattice HP model to describe the PES of a folding protein. Here, each amino acid residue is defined as a single “bead” (i.e., coarse-grained group) which is either hydrophobic or polar. A sequence of such beads represents a simple model of a protein. This same model has been employed in previous studies of protein folding, particularly in the context of global optimization,^35,36^ but also in the study of protein folding.^37^

For our purposes, this simple model has the enormous advantage of being extremely fast to evaluate both potential energy and forces, while still containing the key features of typical protein-folding energetic landscapes. As such, this model provides a route toward validating our graph-driven simulations against direct protein-folding trajectories, as described below.

In all of the following discussion, we use a set of reduced units:

- The mass of each bead is defined to be 1 mu (mass unit).
- The unit of energy ϵ is defined such that the Lennard-Jones well-depth (at equilibrium distance) for interactions between hydrophobic residues is 1 ϵ.
- We use Å as our unit distance.
- Our time is therefore measured in units of .

In the off-lattice HP model employed here, the PES comprises both intramolecular and intermolecular components:
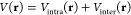
where **r** is the set of 3*N* Cartesian coordinates describing the position of the *N* residues. The intramolecular component, *V*_intra_(**r**), is given by
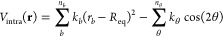
where the first summation runs over all *n*_*b*_ bonds in the protein (formed by adjacent beads), and the second summation runs over all *n*_θ_ bond angles (formed by sequences of three adjacent beads). This intramolecular PES ensures that consecutive beads in the protein chain are held together with a harmonic restraining potential with an equilibrium distance of *R*_eq_ = 3.8 Å and a force-constant of *k*_*b*_ = 3.0 ϵ Å^–2^. Similarly, the bond-angle-bending term (where *k*_θ_ = 0.1 ϵ) serves to prevent the protein artificially “crumpling” to a structure with nonphysical radius-of-gyration. This bond-angle-bending potential is typically a term of the form cos (θ); however, in our simulations, we chose to modify this potential term to the form cos (2θ) instead. This modification artificially “stiffens” the minima of protein-folding intermediates, avoiding generation of trivial pathways in which HP-model proteins fold through a straightforward “collapse” mechanism and enabling our MD and GDS simulations to sample a wider diversity of folding pathways in order to perform a more comprehensive analysis of our contact-map-driven folding scheme.

The intermolecular contribution to the protein PES comprises is a Lennard-Jones potential term that acts on non-nearest-neighbor residues:
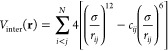
Here, the summation runs over non-nearest-neighbor residue pairs, *r*_*ij*_ is the distance between residues *i* and *j*, and σ = 3.8 Å (representing the effective average radius of each amino acid residue). The factor *c*_*ij*_ is a function of the residue types, as follows (with H for hydrophobic and P for polar):
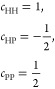
1The choice of the *c*_*ij*_ is clearly designed to reflect the expected folding of the protein structure to obtain preferential packing of hydrophobic residues, and to disfavor hydrophobic–polar residue interactions.

### Molecular Dynamics Simulations

For each protein sequence studied below, we performed extensive MD simulations to generate folding trajectories in the canonical (*NVT*) ensemble. The MD folding trajectories are used below to provide reference folding mechanisms, against which the discretized folding trajectories generated by GDS can be compared.

All MD simulations employed the standard velocity Verlet algorithm for integration. The temperature was controlled using an Andersen thermostat with the coupling constant set as 0.1,^38^ and the positions and momenta of the protein beads were corrected to ensure zero overall linear and angular momenta. A time step of Δ*t* = 0.1 was used in all MD simulations.

For each MD trajectory, we subsequently performed geometry optimization of extracted configurations using the Broyden–Fletcher–Goldfarb–Shanno (BFGS) algorithm.^39,40^ This transforms a MD trajectory, with its inherent thermal fluctuations, into a more coarse-grained representation, describing transport between different local PES minima along the path to the folded structure. For the sequence of local minima visited along each MD trajectory, we can calculate the corresponding contact-map, therefore providing a direct point-of-comparison between our discretized GDS simulations and the MD trajectories (as described further below).

### Double-Ended Graph-Driven Sampling

As noted above, GDS has been used previously to investigate the reactive chemistry of several different systems, including organometallic homogeneous catalysis for hydroformylation reaction and formation of complex organic molecules in the interstellar medium.^23−27^ We have recently reviewed the key features of this approach in the context of chemical reaction network generation;^23^ here, we focus on those aspects most relevant to adapting this simulation strategy to sample candidate protein-folding pathways.

In GDS, the key object of interest is the adjacency matrix **G**(**r**) (or *graph*) describing the current bonding state of the system at configuration **r**. In the context of previous investigations of chemical reactions and catalysis, the adjacency matrix simply describes the bonding between atoms in a binary fashion (with no consideration of bond order). However, in the context of protein folding, for an *N*-bead protein model, **G** is an *N* × *N* symmetric matrix that represents the residue contact-map, describing whether or not a given pair of residues (or beads) form an intermolecular close contact. As such, in the current application to protein folding, the elements of **G** are calculated for any configuration **r** as
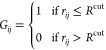
2where *R*^cut^ = 6 Å is the distance cutoff value that represents typical inter-residue close contacts in folded off-lattice HP model proteins (see Figure 3 for an MD verification of this), and *r*_*ij*_ is the distance between beads *i* and *j*.

In GDS, the aim is to generate a directed walk in the discretized space of the contact-map **G**(**r**) that is constructed such that it ensures that a final target folded structure is generated from a given input starting configuration. We begin with input unfolded and target folded protein structures; the generation of these is described below. For these initial and final protein structures, the corresponding contact-maps (**G**^i^ and **G**^f^, respectively) can be straightforwardly calculated using eq 2. Our GDS approach then proceeds to generate a sequence of chemically sensible contact-map transformations that transform **G**^i^ into a graph (or conformation) equivalent to **G**^f^. This sequence of contact-map changes can be viewed as a discretized trajectory that connects the initial (unfolded) protein to the final (folded) state. As described below, postanalysis of these GDS folding paths enables ranking of the feasibility of different folding mechanisms. In other words, the combination of GDS path generation with energy-based postprocessing provides an alternative route to studying protein folding beyond MD-based schemes.

**Figure 2:**
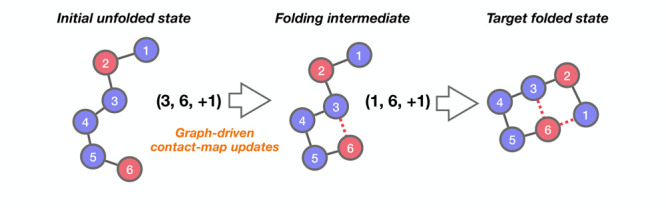
Schematic representation of a two-step folding sequence generated by GDS. Here, a representative protein, modeled as a string of connected hydrophobic and polar “beads” (shown here in red and blue), is folded using two contact-map changes, **C**_1_ = (1, 2, +1) and **C**_2_ = (2, 3, +1), as described in the main text. In this way, a given protein folding trajectory comprising *n*_*r*_ contact-map changes can be readily discretized as a collection of transformations [**C**_1_, **C**_2_, ..., **C**_*n*_*r*__].

In terms of the matrices **G**, any contact-map change, referred to here as the operation **C**, can be characterized by a sequence of three integers, (*k*, *m*, Δ), where (*k*, *m*) are the indices of any two residues in the protein (subject to |*k* – *m*| > 2 to avoid steric clashes) and Δ = ±1 is the proposed *change* in the contact-map matrix-element *G*_*km*_. For example, as shown in Figure 2, contact-map changes (1, 2, +1) and (2, 3, +1) suggest a sequence of changes to inter-residue contacts between residues 1 and 2, then 2 and 3, respectively. As such, sequences of contact-map operations **C** can straightforwardly represent protein-folding trajectories.

Importantly, in the discretized space represented by the contact-map **G**, any folding mechanism that transforms the initial contact-map **G**^i^ into a target contact-map **G**^f^ can be viewed as a sequence of individual contact-map transformations. The goal of GDS is then to identify sequences of operations [**C**_1_, **C**_2_, ..., **C**_*n*_*r*__] that definitively transform **G**^i^ into **G**^f^ after *n*_*r*_ reaction steps. To achieve this, we first note that application of any *n*_*r*_ contact-map changes (or “reactions”) to the initial contact-map **G**^i^ generates a new graph, **G̃** as follows:
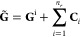
3Here, by definition, the update operation **C**_*i*_ = (*k*_*i*_, *m*_*i*_, Δ_*i*_) acts to modify the element *G*_*k*_*i*_*m*_*i*__ such that *G*_*k*_*i*_*m*_*i*__ → *G*_*k*_*i*_*m*_*i*__ + Δ_*i*_.

The choice of *n*_*r*_ acts as a regularization parameter in our approach. We note that the larger the value of *n*_*r*_, the more complexity we allow within our GDS-generated folding paths. To find a suitable choice of *n*_*r*_ in the simulations reported below, we performed test simulations with large *n*_*r*_ values (typically of the order *N*^2^, for an *N*-bead HP protein model), and gradually lowered *n*_*r*_ to reduce the overall path-length while still generating visually similar folding paths. The results presented below, in comparing MD-generated folding pathways to those given by our GDS strategy, indicate that appropriate *n*_*r*_ values were selected in this way; however, we note that an alternative GDS approach, in which *n*_*r*_ is not fixed at the outset but is instead treated as a variable parameter within our optimization procedure, would also be worth exploring for future work.

To identify discrete folding trajectories in contact-map space we seek to identify sequences of *n*_*r*_ operations, [**C**_1_, **C**_2_, ..., **C**_*n*_*r*__], that minimize the effective distance between the target contact-map (**G**^*f*^) and the contact-map generated by a proposed sequence of transformations (**G̃**). In other words, we seeks to identify contact-map changes **C** such that an optimization function *F* is minimized, where
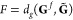
4and *d*_*g*_ is a distance measure capturing the difference between **G**^*f*^ and **G̃**. For example, in our previous work on reaction discovery, we have used a simple element-wise comparison of bonding graphs as an effective distance, defined as
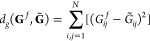
However, this optimization function is not always a suitable choice, especially in systems where the permutational invariance of atoms or molecules is important. In such cases, modification of *F* to account for permutational symmetry is preferred.^24^

In this article, we introduce a further modified version of *F* to account for the different target application in this case, namely protein folding, compared to our previous work on chemical reaction discovery. Here, we focus on characterizing the similarity of two different contact-maps using the hydrophobic coordination number, ϕ(**G**), defined as
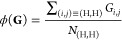
5where *N*_(H,H)_ is the number of pairs of hydrophobic residues. This can be used to calculate the effective distance between two contact-maps as
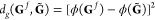
These definitions imply that contact-maps with the same hydrophobic packing are treated the same, regardless of their exact graph. This reflects the fact (observed in our MD simulations below) that a given protein structure will fold to one of a set of closely related structures that only differ through small permutations of hydrophobic residues buried within the core of folded protein structures. However, we emphasize that the choice of the optimization function is somewhat open to modification, depending on the simulation target. For example, we could have equivalently used eq 4 with a distance metric that only depends on the elements of the contact-maps, or we could have employed a distance metric based on other structural parameters such as radius-of-gyration. In fact, as discussed below, the flexibility in the choice of *F* introduces some interesting proposed future applications of GDS, such as explicitly targeting formation of misfolded protein structures.

To identify sequences of contact-map changes [**C**_1_, **C**_2_, ..., **C**_*n*_*r*__] that lead to generation of the folded protein structures, we employ simulated annealing (SA). Here, the discrete space of chosen operations {**C**} is searched using a standard SA algorithm. At each iteration, a random update is made to the sequence of operations [**C**_1_, **C**_2_, ..., **C**_*n*_*r*__]. This update consists of replacing (randomly) up to six different operations at a time. Here, operations are randomly selected and replaced with a new operation acting on a new pair of beads. Uniform probability distributions are used for selection of operations and bead-pairs to replace. If a given proposed change is not consistent with the current contact-map (for example, suggesting a trial move that seeks to form a new inter-residue contact between beads that are already in contact), the move is simply rejected and a new trial move is suggested. After updating the contact-map operation list {**C**}, the new contact-map **G̃** is determined and the optimization function *F* (eq 4) is updated. The new sequence of operations is then accepted or rejected based upon the standard Metropolis criterion, where the probability of acceptance is
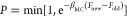
with , and *T*_MC_ serves as a fictitious temperature that is gradually lowered over the course of the SA iterations to drive the optimization process. The final optimized sequence of contact-map operations then represents a folding trajectory in a discretized space.

It is important to note that this optimization procedure, including evaluation of *F*, takes place entirely in the discrete space of the contact-map **G**. As such, the optimization is typically very fast (e.g., less than a minute on a standard desktop computer), such that large numbers of folding trajectories can be generated and ranked in order to build up a picture of the variability of folding pathways for a given protein. However, a significant computational burden is incurred by the postprocessing analysis of the kinetic and thermodynamic characteristics of each folding path, the first step of which requires generation of “real-space” protein structures. We now turn to describing this important step.

### Transforming from Contact-Map Sequence to Residue Coordinates

Following previous implementation of GDS in studying chemical reactions, we use the concept of a *graph restraining potential* (GRP) in order to generate Cartesian-space conformations that directly correspond to the contact-maps (or adjacency matrices) found in the optimized GDS sequences. We note that each optimized GDS trajectory is defined as a sequence of *n*_*r*_ contact-map updates, {**C**}. After each update, we can use the GRP method described here to generate a “real-space” configuration of the protein. Clearly, a sequence of *n*_*r*_ such updates represents a folding trajectory that can subsequently be postanalyzed to reveal key thermodynamic and kinetic features, as discussed below.

The GRP, labeled *W*(**r**, **G**), is an arbitrary function of both residue coordinates and a target graph **G**. The key feature of the GRP is that it should be minimized (ideally obtaining a value of zero) only if the configuration **r** exactly reproduces the target graph **G**. As such, given a target graph **G** and an initial configuration, the coordinates **r** can be optimized under the action of *W*(**r**, **G**) to generate a configuration that is consistent with the target **G**. For GDS, which provides a sequence of *n*_*r*_ graphs representing a folding trajectory, sequential optimization under *W*(**r**, **G**) will therefore generate a Cartesian-space representation of the corresponding folding path.

In our previous work using GRPs to generate intermediate chemical structures in catalytic cycles,^26^ we have found that optimization on the GRP alone can result in creation of highly distorted structures. To circumvent this problem, we propose here a slightly modified version of this GRP approach. In particular, to generate intermediate protein structures corresponding to a particular contact-map given in a GDS trajectory, we perform geometry optimization (using the QuickMin algorithm^41^) on a PES that is the sum of both the off-lattice HP model potential and the GRP:
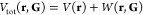
Here, the GRP is given by a pairwise sum over residues:
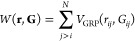
where
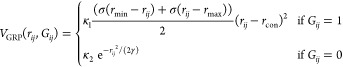
6Here, σ(*x*) is the logistic function, κ_1_ = 1 ϵ Å^–2^, κ_2_ = 6 ϵ, and γ = 6 Å^2^. These values were chosen following some initial trial-and-error geometry optimizations. A deep study on what “optimal” parameters to use was avoided, due to the simple fact it is unnecessary for a bench-marking exercise, provided the method is shown to work. The parameters *r*_min_ and *r*_max_ are the lower and upper thresholds for residue close-contacts, chosen to be 4 and 6 Å, respectively, and  is the midpoint inter-residue distance used as a representative target value for contact-distances. Overall, eq 6 acts to enforce the contact-map **G** on the coordinates **r**. The first (harmonic) term acts to maintain contacts for which *G*_*ij*_ = 1, whereas the second (repulsive) term acts to keep apart residues with *G*_*ij*_ = 0.

After structure optimization on *V*_tot_(**r**, **G**), we proceed with further geometry optimization on the off-lattice HP model PES *V*(**r**) only. This results in a final optimized geometry for the given folding intermediate structure defined through the contact-map **G**. We add that cooperativity effects are implicitly included in our approach by adding the protein potential energy function to the GRP when performing graph-to-coordinate conversions. Our philosophy was that the GRP acts to enforce certain contacts being made in intermediate structures, but the interactions provided by the protein’s potential energy surface then drives the optimized intermediate structures to adopt minimum-energy configurations that naturally account for the relevant cooperative effects. Applying this optimization procedure to each of the intermediate contact-maps generated along a GDS folding trajectory then generates a sequence of *n*_*r*_ intermediate structures which can subsequently be used for further analysis of kinetic characteristics; this is described in the next section.

### Analysis of Folding Paths

For each of the *n*_*r*_ reactions in each GDS trajectory, the optimization on *V*_tot_(**r**, **G**) described above enables generation of Cartesian coordinates for the “reactant” and “product” protein structures. In order to access kinetic information (namely activation energy) for each reaction, we subsequently performed climbing-image nudged elastic band (CINEB) calculations.^28−30^ Here, we use the image-dependent pair-potential (IDPP) method^30^ to generate initial MEP approximations. The CINEB optimization is performed using the QuickMin algorithm, with a target root-mean-square convergence criteria of 5 × 10^–4^ on the perpendicular forces along the CINEB path. All CINEB calculations employed 5 images along the MEP; this choice is motivated by the larger number of CINEB simulations required to generate MEPs for all GDS trajectories.

To validate and quantify how close our GDS folding paths are to those found by MD, we need a metric on the space of trajectories. The choice of the metric is quite important, because it must account for different time alignments of these path (in the sense that different folding “events” may occur at different points along GDS and MD trajectories), while also satisfying the requirement of being insensitive to parametrizations such as MD time steps.

The discrete Frechet distance^31^ satisfies these requirements. Consider two sequences of structures or geometries to be compared (in this case, MD and GDS trajectories). We consider all possible matches between these two sequences that preserve the order of the events. We denote this set of possible matches as Γ. Matchings belonging to Γ are more clearly understood when considering the sequences (*a*_0_, *a*_1_) and (*b*_0_, *b*_1_, *b*_2_) . The matching {(*a*_0_, *b*_0_), (*a*_0_, *b*_1_), (*a*_1_, *b*_2_)} ∈ Γ is valid while {(*a*_0_, *b*_0_), (*a*_1_, *b*_1_), (*a*_0_, *b*_2_)} ∉ Γ is not, since we go backward along the *a*_0_ sequence. The discrete Frechet metric is then computed by finding the optimal matching, such that maximum deviation (which is chosen as the distance between conformations, see below) along the matched path is minimized. In other words, for given sequences *A* = (*a*_*n*_), *B* = (*b*_*m*_), a matching can be thought of as a reparametrization of the sequences to *A*′ = (*a*_*n*_*i*__), *B*′ = (*B*_*m*_*i*__) where *m*_*i*+1_ = *m*_*i*_ or *m*_*i*+1_ = *m*_*i*_ + 1 to preserve time-ordering. The discrete Frechet metric is then
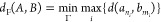
7where *d* is a suitable metric comparing individual structures.

The Frechet metric has been previously shown to be able to capture differences and similarities in paths generated by various MD codes.^31^ In our trajectories, the difference *d*(*a*_*n*_*i*__,*b*_*m*_*i*__) between conformations along trajectories is measured as the root-mean-square deviation (RMSD) of the set of inter-residue distances between two different conformations, thereby avoiding the need for rotational and translational alignment of protein structures.

## Application, Results, and Discussion

The central goal of this paper is to demonstrate that GDS can generate an ensemble of physically sensible protein-folding trajectories that can be used to further analyze thermodynamic and kinetic characteristics. To achieve this goal, we must (i) demonstrate that the GDS-generated paths are comparable to folding paths generated by brute-force MD simulations, and (ii) show how GDS-generated paths can be ranked to identify the most relevant folding trajectories on the basis of physical characteristics. The first target here provides a route to validating the physical correctness of GDS-generated paths, while the second target provides a route to ranking and selecting the “most relevant” GDS folding paths based on the characteristics of the paths alone (i.e., without requiring MD reference trajectories for comparison).

### Protein Folding with *N* = 13 Residues

To begin, we present a detailed description of our MD and GDS results, as well as their cross-validation, for an HP-model protein with *N* = 13 residues. The particular sequence of hydrophobic (H) and polar (P) residues chosen in this case corresponds to one of the previously studied Fibonacci sequences: HPPHPPHPHPPHP (or (HPP)_2_(HP)_2_PHP). This class of HP proteins are defined recursively, where the *n*th Fibonacci protein is defined as the polymer formed when attaching the (*n* – 1)th Fibonacci protein to the end of the (*n* – 2)th one; the first two sequences are defined simply to be H and P, respectively. This class of sequences has been regularly studied using simple protein models,^35−37^ and we adopt these sequences here to enable comparison to this previous work.

#### Generation of MD Benchmark Data

We began by using MD simulations to generated benchmark folding trajectories against which GDS could be compared and validated. First, we sought to identify both the target folded state and an appropriate simulation temperature for modeling folding of this *N* = 13 protein. The target folded state was required for the subsequent GDS simulations, while an appropriate temperature is required to generate MD folding trajectories that visit well-defined sequences of local minima along the folding path. For the simple model proteins studied here, high temperatures are sufficient to quickly drive the folding process to completion without trajectories spending significant residence time in local minima along the folding path. This contrasts with the typical “folding funnel” picture and prevents adequate comparison to our GDS folding paths. Instead, the MD simulation temperature must be chosen such that the protein folds over an appropriate simulation time scale, but at the same time spends sufficient time in local minima to enable their clear identification and comparison to the intermediates generated by GDS. We emphasize here that this requirement on the MD folding trajectories is only a requirement for validation of GDS paths; artificially “slowing down” the MD folding dynamics enables more straightforward validation of GDS folding trajectories.

To identify an appropriate MD simulation temperature, we performed *NVT* MD simulations at temperatures of *T* = [0.1, 0.125, 0.175] reduced units. At each temperature, 480 MD trajectories of total time *t* = 1000 (reduced units) were performed, using a time step of 0.1 reduced time units. We periodically calculated the average distance between all pairs of hydrophobic “beads” in the protein on each trajectory. From prior analysis of MD folding trajectories, it was clear that folded structures adopted conformations in which hydrophobic beads packed into protein interior, with polar residues on the exterior surface, as one would generally expect for these simple HP model proteins. As such, a histogram of the distances between hydrophobic beads would be expected to exhibit different peaks, corresponding to different intermediates along the protein folding path at different times. In the long-time limit, the inter-hydrophobic bead distance corresponding to the folded structure will become evident in these plots.

**Figure 3:**
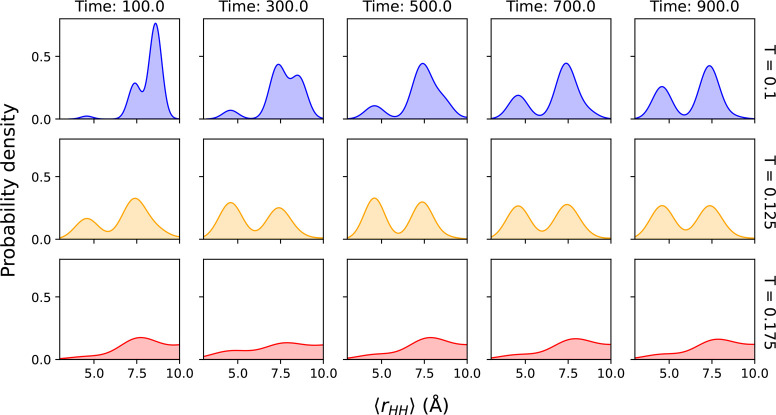
Time evolution of average inter-hydrophobic-residue distances at different temperatures (indicated the on right-hand side) and times (indicated along top). Analysis of protein structures generated by MD suggest that conformations with average inter-hydrophobic distances ⟨*r*_HH_⟩ ≤ 6 represent the folded state.

This expected behavior is confirmed in Figure 3, which shows probability density of average inter-hydrophobic-residue distances as a function of time and temperature. At the highest temperature considered here (*T* = 0.175), the histograms at all times show a broad distribution of hydrophobic-residue distances with poorly defined peaks. Furthermore, the relative changes over time are quite small, in the sense that the broad distance distribution is observed at quite early simulation times and remains similar throughout the rest of the simulation. From visual analysis of the corresponding MD trajectories, it is found that the peak at a distance ⟨*r*_HH_⟩ ≤ 6 represents the folded protein conformation, with a dense core of hydrophobic residues surrounded by exterior polar residues (also shown in Figure 1). In this high-temperature trajectory, significant probability density is found in this peak at even the shortest time (*t* = 100), indicating that the folded state forms rapidly. As such, in terms of comparing to GDS trajectories, where our requirement is to have a set of well-defined intermediates which are occupied as discrete states along the trajectory, this high-temperature MD simulation is not appropriate for benchmarking.

At the intermediate temperature *T* = 0.125, qualitative differences are observed, with much better defined peaks, demonstrating that the MD trajectories exhibit behavior more akin to visiting discrete minima on the PES. However, even for this lower temperature, it is found that the folded state can again form early in the simulation, as demonstrated by the significant peak at ⟨*r*_HH_⟩ ≤ 6 in Figure 3 for *T* = 0.125 and time *t* = 100. Again, these simulations demonstrate that this temperature is not appropriate for benchmarking GDS.

At the lowest temperature considered (*T* = 0.1 reduced units, Figure 3), it is clear that the folding dynamics is effectively slowed, but the folding state can still be successfully and reliably generated. In fact, we find that 196 MD trajectories (out of 480) at this temperature reached the folded state (defined hereafter as structures with average inter-hydrophobic-residue distance ⟨*r*_HH_⟩ ≤ 6). However, most importantly from the point-of-view of benchmarking GDS trajectories, we find that the folding dynamics at *T* = 0.1 are representative of trajectories that visit different PES local minima on the approach to the folded state, while also exhibiting sufficient residence time in each to enable ready identification and comparison to GDS. This is clear from the fact that the peak representing the folded state at ⟨*r*_HH_⟩ ≤ 6 increases gradually over the course of the entire simulation time, demonstrating that folded structures are formed over a range of time scales (rather than the much faster folding time scales observed for *T* = 0.175 and *T* = 0.125). Furthermore, the appearance of different peaks with different ⟨*r*_HH_⟩ throughout the MD simulations at *T* = 0.1 further indicates exploration of different minima on the PES on the path to the folding state. Overall, therefore, we use MD simulations at *T* = 0.1 reduced units as benchmark data for validation of GDS in this section.

#### Validation of GDS Trajectories

We now turn to describing generation and validation of GDS trajectories. Here, we performed 480 GDS simulations with a maximum of *n*_*r*_ = 22 contact-map updates and fictitious temperature for optimization starting at *T*_MC_ = 250 reduced units and linearly decreased to zero over the course of 10^5^ Monte Carlo updates. We note that this limit indirectly biases GDS toward sampling paths with lower complexity, and can be adjusted to study more exotic paths (which are more unlikely to occur). In total, 433 of the 480 paths ended at a protein structure with an average inter-hydrophobic-residue distance *r*_HH_ ≤ 6; these were selected as paths for further analysis. Given that this system is strongly characterized by the number of hydrophobic residues in contact, we focus on using the hydrophobic coordination number ϕ(**G**) (eq 5) to represent different configurations.

**Figure 4:**
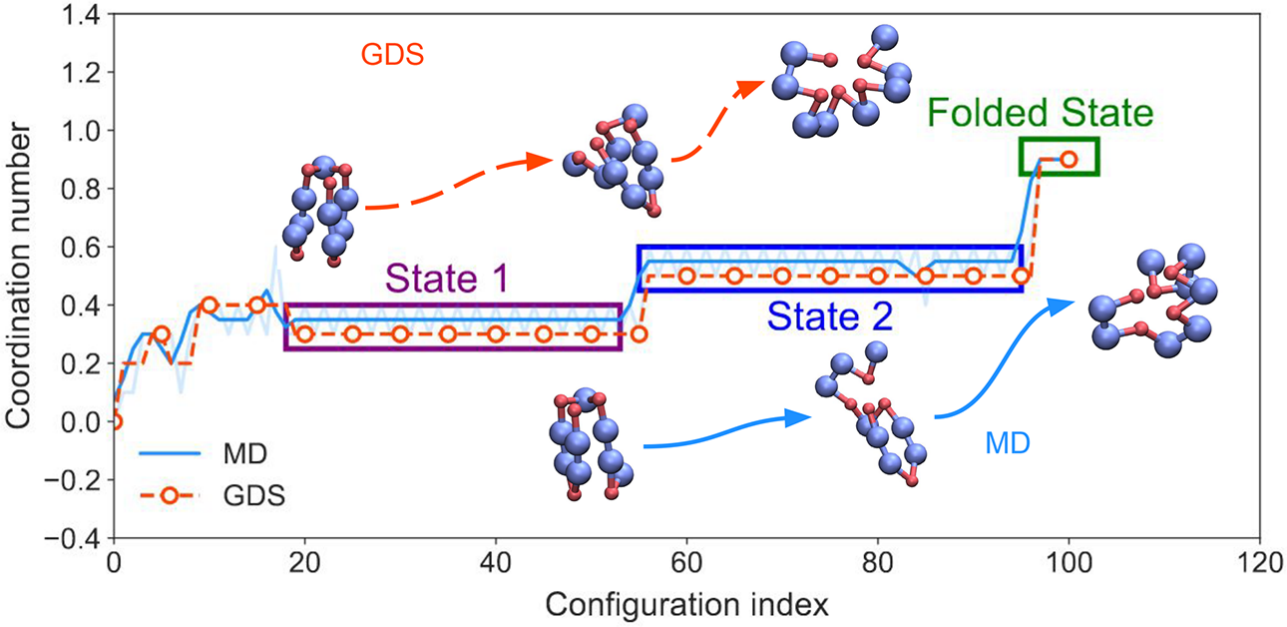
Comparison of aligned configurations obtained from a representative MD and GDS trajectory. Here, the Frechet distance metric was used to identify closely matching configurations along MD (blue) and GDS (red) trajectories. For the MD trajectories, this results in selection of configurations that can undergo small fluctuations in coordination numbers (as shown in the fainter blue line). Local windowing over these configurations removes these thermal conformational changes and makes comparison to the GDS configurations more straightforward. The coordination number ϕ (eq 5) is shown for the aligned configurations. Both the MD and GDS trajectories in this case fold through two intermediate states. The inset sequences of structures show configurations for states 1 and 2, as well as the final folded state, obtained from GDS (upper) and MD (lower), respectively.

Figure 4 shows a side-by-side comparison of representative folding trajectories obtained by MD (with local structure optimization) and GDS. We note that, in terms of total number of configurations, the MD trajectory is much longer than the GDS trajectory. This is expected because GDS views folding trajectories as a series of “hops” between graphs, whereas MD trajectories can spend a significant time in a single local minimum. In Figure 4, the MD and GDS trajectories have been aligned using the Frechet distance metric. As a result, the progress coordinate represents a sequence of aligned conformations along the GDS and MD trajectories (rather than a physical time). In the particular example of Figure 4, both the MD and GDS trajectories exhibit two intermediate states on the folding path, before reaching the final folded state. Structures along the MD and GDS paths (insets, Figure 4) are not identical (and neither are they expected to be due to the different methods of generating these paths) but demonstrate structural similarities in regard to the packing of the hydrophobic core. Ultimately, it is clear that both MD and GDS converge to a folded structure with essentially identical hydrophobic-residue coordination number; visualization demonstrates the clear similarities of these folded states.

**Figure 5:**
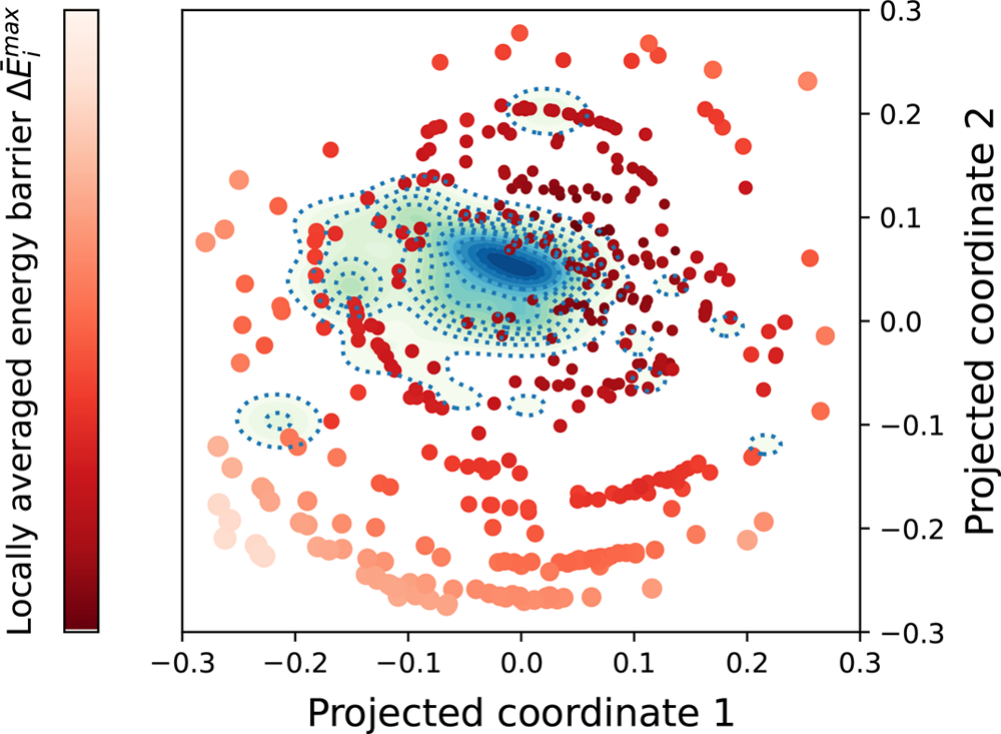
Multidimensional scaling (MDS) projection of GDS and MD trajectories for *N* = 13 model protein. Pairwise distances between entire trajectories are calculated using the Frechet distance. Kernel density estimation was used to represent the MD trajectory distribution, with each GDS trajectory represented as a single point, colored and scaled proportionally to the locally averaged activation barrier (see text for description).

The most important question to address here is whether the GDS folding trajectories are representative of the same folding paths generated directly by *NVT* MD simulations; this validation is the key goal of this article. To compare these two different simulation approaches, Figure 5 presents a comparison of GDS trajectories and MD folding trajectories in a reduced-dimensional space. Here, we have used multidimensional scaling (MDS)^32,33^ to project from the high-dimensional space of MD and GDS trajectories onto two reduced-dimensional coordinates. This projection is constructed such that pairwise distances between points in the full-dimensional space (i.e., entire trajectories) are preserved in the lower-dimensional 2D projection. As noted in Figure 1, to enable comparison of MD trajectories (which contain one configuration at each time-step along the trajectory) and GDS folding paths (which typically contains far fewer intermediates along the folding paths than a corresponding MD trajectory), we employed a two-step process. First, every structure in each MD trajectory was energy-minimized using the L-BFGS algorithm; this has the effects of removing minor thermal fluctuations and collapsing configurations onto the nearest local PES minima. Second, the pairwise distance between any pair of trajectories (either MD or GDS) was subsequently calculated using the Frechet metric described above; this offers a convenient comparison between different trajectories, regardless of how many intermediate structures a given folding pathway visits. Furthermore, we also note that the Frechet metric represents a “time-ordered” comparison, such that trajectories labeled with a high degree of similarity must visit similar sets of intermediate structures in a similar order on the path to the folded state.

Figure 5 shows the results of the MDS analysis for MD and GDS folding trajectories. The MD simulations are represented as a continuous probability distribution, obtained using kernel density estimation (KDE) based on the MDS projection coordinates.^40^ This is done simply as a visual aid to better understand the distribution of the GDS and MD trajectories. Each GDS trajectory is represented as a single-point, colored according to its maximum activation barrier (as determined by CINEB calculations for all intermediate contact-map changes). Importantly, we find that a significant number of the GDS trajectories overlap with the MD density distribution - demonstrating that GDS can indeed sample folding trajectories that are representative of those generated by direct MD. In addition, we note that GDS generates a much wider distribution of folding trajectories than MD simulations. This is expected to be the case because the GDS path-generation procedure is not directly biased toward generating folding paths with specified thermodynamic and/or kinetic characteristics in the same way that MD simulations are. For example, in the MD simulations, the impact of temperature is to bias the sampled folding paths to those which contain energetic barriers that are on the order of a few multiples of the available thermal energy. However, in the case of GDS, no such implicit biasing exists, leading to sampling of a broader range of folding paths. Finally, as would be expected, we find that those GDS paths with lower maximum activation energies (hence, overall kinetically favored) overlap most closely with the MD-generated trajectories, with GDS trajectories possessing higher activation energy lying outside the main domain of the MD density distribution.

From Figure 5, we can conclude that, based on the MDS comparison using time-ordered Frechet distance, GDS can indeed generate protein-folding trajectories which map onto those that can be generated by direct MD simulations; this is one of the main findings of this article. Furthermore, we note that visualization of the MD and GDS trajectories demonstrates mechanistic similarities in the folding paths obtained by these different methods, as is also emphasized in Figure 4.

#### Independent Ranking of GDS Trajectories

Above, we have demonstrated that GDS can generate protein-folding paths that closely resemble those that are generated by direct MD simulations. While this is satisfying, the comparison of GDS and MD only serves the purpose of validation in this case. For a more general protein-folding problem, it may actually be impossible to generate a satisfactory folding trajectory using MD, due to the well-known time scale challenge; in this case, validation of GDS paths against MD trajectories would be impossible. As such, it is crucial to be able to rank the physical accuracy of different proposed GDS paths *without reference to benchmark MD trajectories*. This would enable one to use GDS to study protein-folding paths independently, without requiring reference to MD trajectories. In this section, we discuss how this GDS ranking can be achieved.

Our approach here is to define a suitable metric that can be used to rank GDS folding paths without requiring reference to MD trajectories. However, to ensure that this ranking is physically sensible, we clearly demonstrate here that our proposed GDS ranking successfully aligns with MD-based predictions.

For the purposes of this discussion, we assume that we have generated a large number of GDS trajectories that reach to the target folded state. Furthermore, we assume that we have successfully performed NEB refinement of all of the MEPs connecting intermediates along the GDS paths. We then make the simple assumption that the most relevant folding pathways are those which have the lowest *maximum* activation energy along the GDS trajectory. The clear basis of this is that the largest barrier has the largest impact on the overall folding kinetics, as well as the standard assumption that folding trajectories would naturally be expected to follow the “path of least resistance” in regard to intermediate energetic barriers.

The argument above suggests that a suitable measure for ranking GDS is simply the highest energetic barrier, Δ*E*^max^, determined by NEB for all intermediate transitions along the entire GDS trajectory. However, in practice, challenges associated with NEB calculations can have a significant impact if this straightforward ranking is used. In particular, poorly converged NEB calculations, as well as slight differences in conformations of transition end-points, can lead to quantitatively different Δ*E*^max^ for trajectories that appear, by other measures (such as Frechet distance), to be similar.

Instead, we find that local averaging (or smoothing) over closely related GDS trajectories serves to remove these errors to provide a more meaningful ranking metric. In particular, for a given GDS trajectory *i*, we calculate a locally averaged maximum energy, Δ*E̅*_*i*_^max^, as
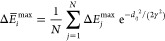
8Here, Δ*E̅*_*i*_^max^, the locally averaged maximum barrier height for GDS trajectory *i*, is given as a weighted-sum over the maximum energetic barriers Δ*E*_*j*_^max^ for all *N* GDS trajectories. The weighting function here is simply taken to be a Gaussian function of the Frechet distance *d*_*ij*_ between trajectories *i* and *j*.

Equation 8 offers a simple ranking metric that can be calculated for any GDS trajectory without reference to MD simulations. This metric can be used to rank the plausibility of different GDS folding paths. However, we also need to confirm that this ranking metric is sufficient to *accurately* rank GDS folding mechanisms. In other words, we need to confirm that GDS trajectories that are flagged as highly ranked using the ranking metric of eq 8 do indeed correlate closely with folding trajectories generated by MD simulations.

**Figure 6:**
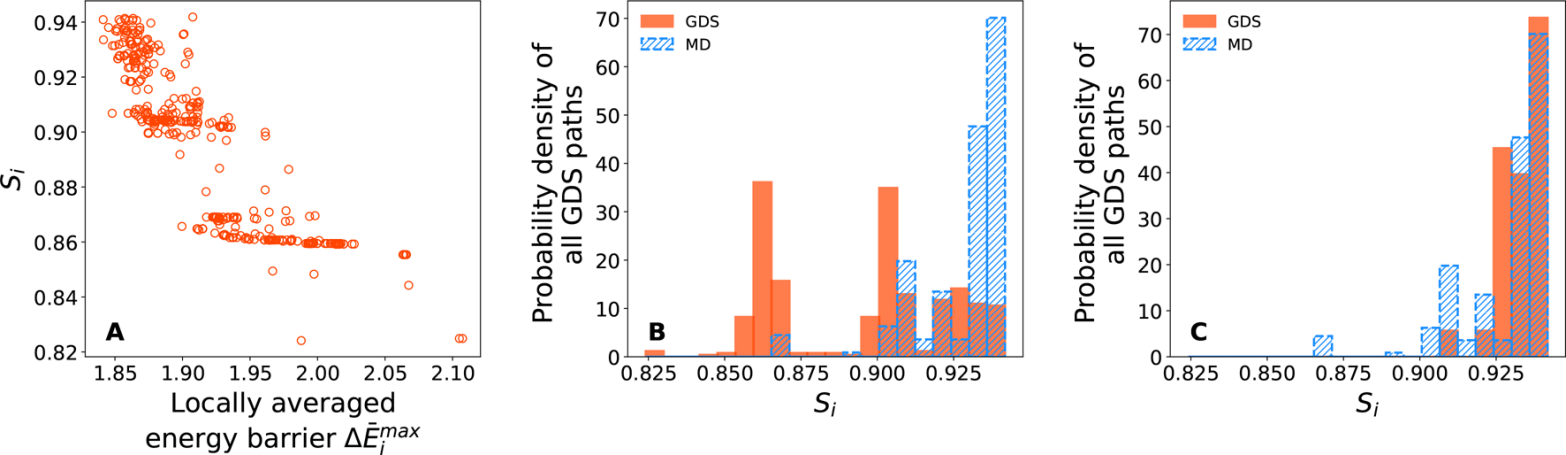
Plots demonstrating ranking of GDS paths for *N* = 13 protein model. (A) Correlation between locally averaged maximum energy barrier Δ*E̅*^max^ and locally averaged similarity to MD trajectories. As expected lower Δ*E̅*^max^ correspond to GDS trajectories which are more similar to MD-generated trajectories. (B) Distribution of similarity scores *S*_*i*_ determined for all MD and GDS trajectories. (C) Distribution of similarity scores for the 30 GDS paths with the lowest Δ*E̅*^max^.

To assess this correlation, Figure 6 plots the GDS ranking metric *E̅*_*i*_^max^ from eq 8 and a path-similarity score that represents the similarity of GDS trajectories to MD trajectories. We emphasize that the GDS path ranking of eq 8 does not rely on availability of MD simulation trajectories (and so is applicable to examples when MD cannot satisfactorily fold a protein within a given simulation time). In particular, for any GDS trajectory *i*, we calculate a path-similarity score as
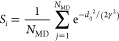
9where *d*_*ij*_ is the Frechet distance between GDS trajectory *i* and MD trajectory *j*. As in the case of eq 8, the similarity score of eq 9 represents a locally averaged comparison of each GDS trajectory to the local neighborhood of MD trajectories. If a given GDS trajectory has a large similarity score *S*_*i*_, this implies that the GDS trajectory is similar to one or more MD trajectories and hence represents a physically plausible folding path.

If the GDS ranking of eq 8 is capable of successfully identifying GDS trajectories that are most physically plausible (in the sense that they are most similar to MD folding trajectories), then we would expect to see a strong correlation between Δ*E̅*_*i*_^max^ and *S*_*i*_. Somewhat satisfyingly, this is exactly what is observed in Figure 6a. We see a clear correlation between Δ*E̅*_*i*_^max^ and *S*_*i*_, with the better GDS trajectories with lower Δ*E̅*_*i*_^max^ corresponding the larger similarity scores *S*_*i*_. This demonstrates that the ranking metric Δ*E̅*_*i*_^max^ is capable of identifying physically sensible GDS trajectories that would be expected to match those generated by MD simulations. This is an important conclusion; GDS simulations, combined with ranking based on Δ*E̅*_*i*_^max^, can be used to generate MD-like trajectories for further analysis, offering a new route to studying long time scale processes such as protein folding.

One technical note here is the choice of the length scale, γ (eqs 8 and 9). Here, we chose γ = 1 but also found that the qualitative features of Figure 6a are quite insensitive to this choice. We note that a robust alternative to NEB calculations could make the locally averaging procedure redundant, as discussed below.

While Figure 6A successfully demonstrates that “physically plausible” GDS trajectories (i.e., in the sense of matching MD-generated trajectories) can be generated and selected, Figure 6B,C further underlines this point. Here, we consider computing the similarity score *S*_*i*_ for both MD and GDS trajectories to determine if the GDS trajectories selected based on Δ*E̅*^max^ have a systematically lower *S*_*i*_ or not. In Figure 6B, showing the distribution of *S*_*i*_ for all MD and GDS trajectories, we see that the MD trajectories exhibit high self-similarity (as expected), with the GDS trajectories distributed over a wider similarity range. However, in Figure 6C, we compare the similarity distributions for MD trajectories to the distribution obtained for the top 30 GDS trajectories when ranked according to Δ*E̅*^max^. In this case, there is a very strong overlap of highly ranked GDS trajectories and MD self-similarity. In other words, Figure 6C further demonstrates that the GDS trajectories with lower Δ*E̅*^max^ look very similar to MD folding trajectories.

To conclude this section, we have successfully demonstrated that high-quality folding trajectories, representative of the folding paths found by MD, can instead be generated by GDS and identified using a simple ranking metric (eq 8). Importantly, this ranking can be performed independently of any MD simulations; in other words, GDS can be used to study protein-folding paths without requiring MD reference trajectories.

### Protein Folding for *N* = 21 and *N* = 34 Residues

In the section above, we have presented a detailed comparison of GDS trajectories and MD folding paths, validating our GDS approach and identifying a route to independently ranking GDS trajectories without reference MD data. In this section, we apply the same strategy to demonstrate applicability of GDS to larger protein systems. Specifically, we repeat the comparison of MD and GDS trajectories for larger *N* = 21 and *N* = 34 protein sequences. These simulations again demonstrate how GDS can be used for plausible folding trajectory generation.

For the *N* = 21 and *N* = 34 proteins, the H/P sequences were chosen to be
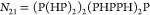

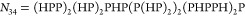
where subscripts indicate repetition of the indicated units. As in the case of *N* = 13, these larger proteins are so-called Fibonacci sequences that have been studied previously. Following on from the discussion about MD trajectories above, the same temperature assessment was performed for both of the larger proteins. In each case, a temperature *T* = 0.125 reduced units was selected, leading to sufficiently “slow” folding dynamics to enable straightforward comparison to GDS. As in the case of the *N* = 13 protein, visual study of the folding trajectories demonstrated that a protein conformation with ⟨*r*_HH_⟩ ≤ 6 corresponded to a compact and stable folded state.

All MD simulations for *N* = 21 were performed in the same way as our previous *N* = 13 simulations. In total, 480 MD trajectories were generated, of which 434 were found to fold successfully. The rise in rate of successful folding in comparison to *N* = 13 can be attributed to the increase of temperature to *T* = 0.125. This was used because *T* = 0.1 MD trajectories were found to be too slow in escaping intermediate minima on the PES. For *N* = 34, we found that *T* = 0.125 resulted in appropriately slow dynamics; however, the MD trajectories required ca. 10000 time steps (instead of ca. 1000 previously) to see enough folded trajectories. In total, 109 of the 480 simulations resulted in folded trajectories for *N* = 21. Due to the length of the trajectories, however, we chose every third time step for computational tractability in further analysis.

For *N* = 21, a total of 960 GDS simulations were performed, with maximum reaction-length *n*_r_ = 50. Out of these, we found that 930 (97%) successfully generated protein-folding trajectories. Similarly for *N* = 34, 858 out of 1440 GDS simulations (60%) generated protein-folding trajectories with *n*_r_ = 50. The decrease in the success rate of GDS in finding successful folding trajectories is clearly related to the increasingly challenging optimization problem as *N* increases. Improvements to our SA-based optimization would clearly be expected to help here, although we note that, even for *N* = 34, GDS can generate large numbers of folding trajectories without further optimization.

**Figure 7:**
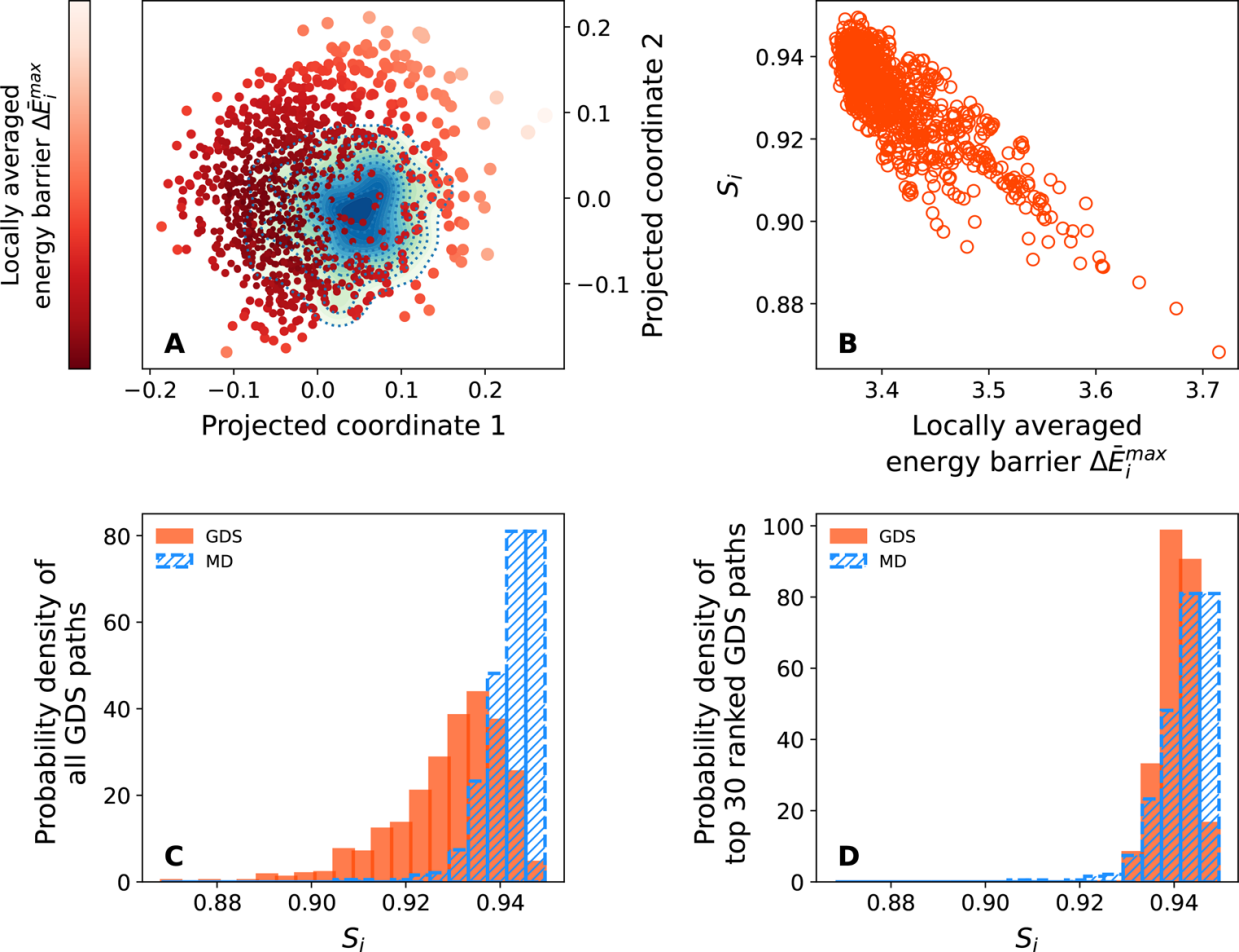
GDS and MD results for protein folding with *N* = 21 residues. (A) MDS for MD trajectories (represented as continuous density distribution) and GDS trajectories (represented as points, colored according to locally averaged maximum barrier height). (B) Correlation between similarity score *S*_*i*_ and locally averaged maximum barrier Δ*E̅*^max^. (C) Similarity score distribution for MD trajectories (blue) and GDS trajectories (red). (D) Similarity score distribution for MD trajectories (blue) and the 30 top-ranked GDS trajectories, as predicted using Δ*E̅*^max^.

Figure 7 compiles the same set of results as Figures 5 and 6, but for the *N* = 21 protein; however, it is clear that the same qualitative trends are observed for *N* = 21 as for *N* = 13. First, in Figure 7A, MDS demonstrates that there is some overlap between the GDS and MD trajectories. As in the *N* = 13 case, many GDS trajectories overlap with the density distribution of the MD trajectories, and it is generally found that those overlapping GDS trajectories also demonstrate lower maximum-energy barriers (with higher maximum-energy barriers sitting on the periphery of the MD distribution). Figure 7B also demonstrates that our ranking criteria of eq 8 work for *N* = 21 too; GDS trajectories with high similarity to MD trajectories exhibit lower Δ*E̅*^max^. As above, this is further confirmed in Figures 7C,D, demonstrating that the entire set of GDS trajectories span a broad range of characteristics (or similarities to MD), whereas the top-ranked GDS trajectories according to Δ*E̅*^max^ are clearly most similar to the MD trajectory set.

**Figure 8:**
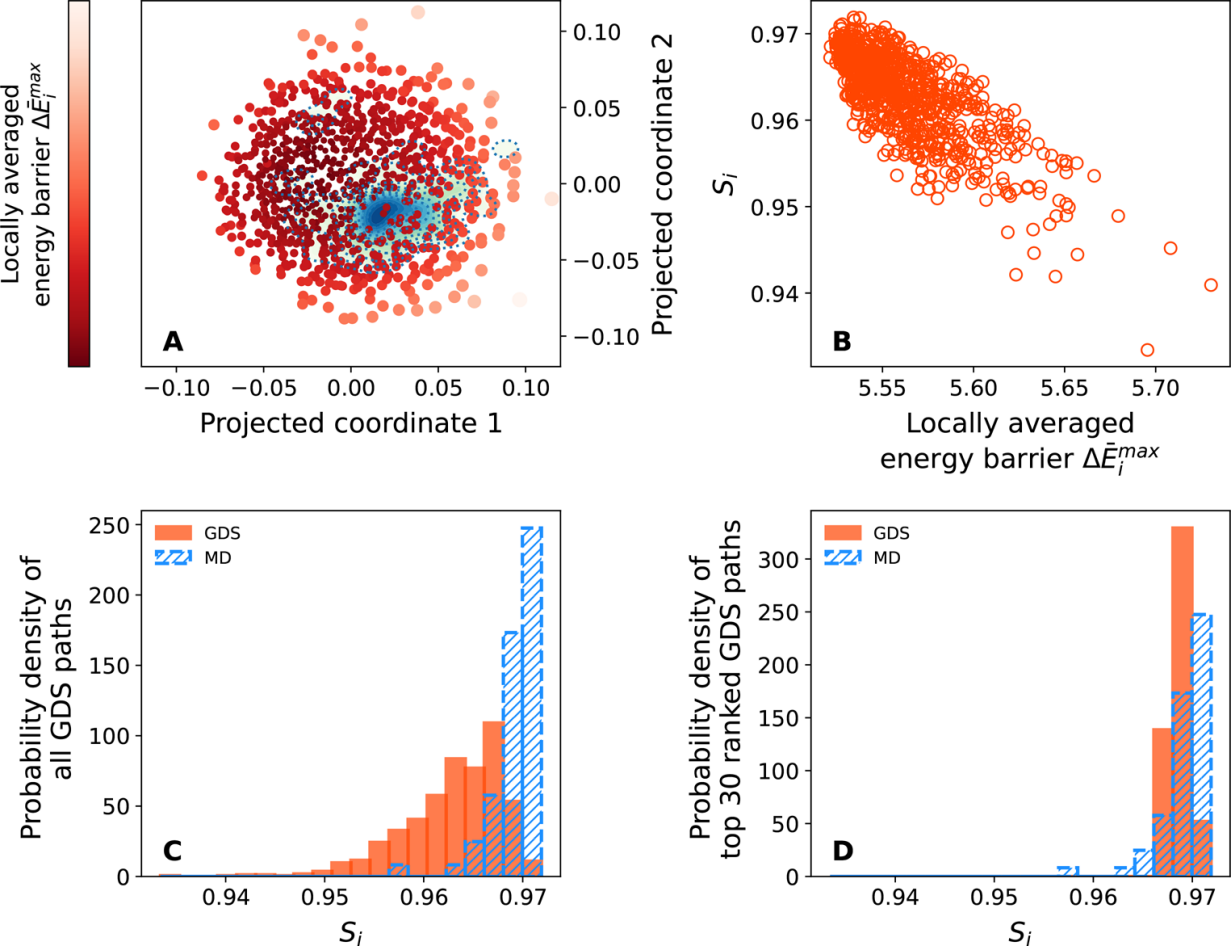
GDS and MD results for protein folding with *N* = 34 residues. (A) MDS for MD trajectories (represented as continuous density distribution) and GDS trajectories (represented as points, colored according to locally averaged maximum barrier height). (B) Correlation between similarity score *S*_*i*_ and locally averaged maximum barrier Δ*E̅*^max^. (C) Similarity score distribution for MD trajectories (blue) and GDS trajectories (red). (D) Similarity score distribution for MD trajectories (blue) and the 30 top-ranked GDS trajectories, as predicted using Δ*E̅*^max^.

Everything that is noted above for the case of *N* = 21 is found to be equally true for the larger *N* = 34 protein, as shown in Figure 8. As confirmed in Figure 8A, GDS trajectories overlap significantly with the MD density distribution after MDS, and Figure 8B confirms good correlation between the similarity *S*_*i*_ and the locally averaged maximum barrier Δ*E̅*^max^. As in the case of *N* = 13 and *N* = 21, Figures 8C,D also firmly demonstrates that GDS can generate a broad range of folding paths, but the best-ranked paths according to Δ*E̅*^max^ are strongly similar to MD folding trajectories.

To summarize, this section has demonstrated that our GDS simulation approach, and the related ranking and postprocessing analysis, is as equally applicable to larger proteins with *N* = 21 and *N* = 34 residues as it was to *N* = 13 proteins. Most importantly for future applications, ranking GDS trajectories based on simple measures such as Δ*E̅*^max^ offers a route to identifying an ensemble of “good” folding trajectories without recourse to MD trajectories for validation.

### Challenges in Further GDS Simulations

Finally, despite the clear success in using GDS to generate protein-folding ensembles above, it is worth highlighting a number of outstanding methodological challenges that we have come across in these simulations. First, it is clear that the current implementation of GDS requires one to perform a large number of NEB-type calculations for all of the intermediate reactions generated for each GDS trajectory. These calculations are necessary to allow ranking of GDS coordinates based on maximum activation energies. The computational demands of these NEB calculations can be high, and clearly increase as the length of GDS-generated paths is increased or as more GDS paths are generated to improve sampling of the folding ensemble. As such, it is clear that steps should be taken to reduce the computational burden of the NEB postprocessing; here, machine-learning (ML) strategies for predicting activation energies given reactant and product configurations may prove useful, as has been recently demonstrated.^42−46^

Second, as well as the required large number of NEB calculations, we have found that NEB calculations can often fail to sufficiently converge or can be unstable for some of the intermediate protein conformational changes generated by GDS. This is found to be particularly true for contact-map changes for protein conformations that already possess quite densely packed hydrophobic cores. As well as deploying ML strategies to address this point, another option is to deploy alternative MEP-finding routines that might be better suited for such systems. Here, the growing-string method^47−49^ may prove useful, given that this approach is less reliant on the availability of an initial MEP guess in the same way that our current IDPP-based implementation of NEB is.

Finally, it is worth noting that neither of these simulation challenges is actually inherent to GDS, but we implicitly rely on NEB-type calculations to obtain MEP information characterizing the kinetics of different folding paths. As such, seeking an optimal combination of GDS and postprocessing strategies is of key importance and will be the subject of our future work.

## Conclusions

In this article, we have introduced a new contact-map-driven approach to generating plausible protein folding trajectories. Given a target folded structure, which may be characterized either as a single specific conformation or through an auxiliary function such as a target coordination number, our GDS strategy can generate ensembles of pathways that lead from the unfolded state to the target folded state through a series of transitions between discrete PES minima. Beyond a function defining similarity to the target folded state, our approach does not require an “order parameter” or “driving coordinate”, and circumvents the time scale problems associated with brute-force MD simulations of protein folding. We emphasize that this function is only evaluated for the final structure generated by a proposed GDS folding path, in order to evaluate similarity with the target folded state (irrespective of the pathway taken to generate the final structure), representing a key advantage over methods that utilize a reaction coordinate requiring some *a priori* knowledge of the nature of the pathway(s) rather than just the final state.

The focus of this article has been on two aspects of the GDS approach. First, we have highlighted how we can validate the “correctness” of the GDS-generated folding trajectories by comparing, where possible, to the folding trajectories generated by direct MD simulations for AB-model proteins. By performing MDS analysis of the MD and GDS folding trajectories, using the time-ordered Frechet metric as a measure of the effective similarity of different folding paths, we demonstrated that GDS can indeed generate folding trajectories that are representative of those generated by direct MD simulations. Second, we have shown how the quality, or physical plausibility, of GDS paths can be ranked without reference to benchmark MD trajectories. In particular, we have suggested a simple metric that can be used to independently rank GDS trajectories. We have shown that highly ranked GDS trajectories, according to our energy-based-ranking metric, correspond to similar folding paths as would be generated by direct MD simulations. This is an important achievement, indicating that GDS simulations can be used to generate protein-folding ensembles *and* rank the physicality of different folding paths.

The validation performed here therefore opens the door to further applications of GDS to problems in protein folding and, more generally, self-assembly. As we have noted above, important challenges to our approach remain, most notably the efficient generation and characterization of MEPs connecting intermediate structures generated along GDS trajectories. We suggest that more refined approaches, such as the growing-string method, will help in this and note that there is the more general opportunity to deploy AI/ML as a route to energy barrier prediction. Of course, the next step in evolution of this strategy will be adaptation to and analysis of folding paths for more realistic protein interaction models, going beyond the simple AB-model. Following the validation exercise performed here, we hope to report further such developments in forthcoming work.

## Data Availability

Data used in generating Figures 5–8 are available through the Warwick Research Archive Portal at wrap.warwick.ac.uk/174756.
